# Epigenetic and Transcriptional Pre-patterning—An Emerging Theme in Cortical Neurogenesis

**DOI:** 10.3389/fnins.2018.00359

**Published:** 2018-05-29

**Authors:** Mareike Albert, Wieland B. Huttner

**Affiliations:** Max Planck Institute of Molecular Cell Biology and Genetics, Dresden, Germany

**Keywords:** gene regulation, histone methylation, neocortical development, neural progenitor cell, Polycomb, epigenetics, chromatin, neurogenesis

## Abstract

Neurogenesis is the process through which neural stem and progenitor cells generate neurons. During the development of the mouse neocortex, stem and progenitor cells sequentially give rise to neurons destined to different cortical layers and then switch to gliogenesis resulting in the generation of astrocytes and oligodendrocytes. Precise spatial and temporal regulation of neural progenitor differentiation is key for the proper formation of the complex structure of the neocortex. Dynamic changes in gene expression underlie the coordinated differentiation program, which enables the cells to generate the RNAs and proteins required at different stages of neurogenesis and across different cell types. Here, we review the contribution of epigenetic mechanisms, with a focus on Polycomb proteins, to the regulation of gene expression programs during mouse neocortical development. Moreover, we discuss the recent emerging concept of epigenetic and transcriptional pre-patterning in neocortical progenitor cells as well as post-transcriptional mechanisms for the fine-tuning of mRNA abundance.

## Introduction

**Figure 1:**
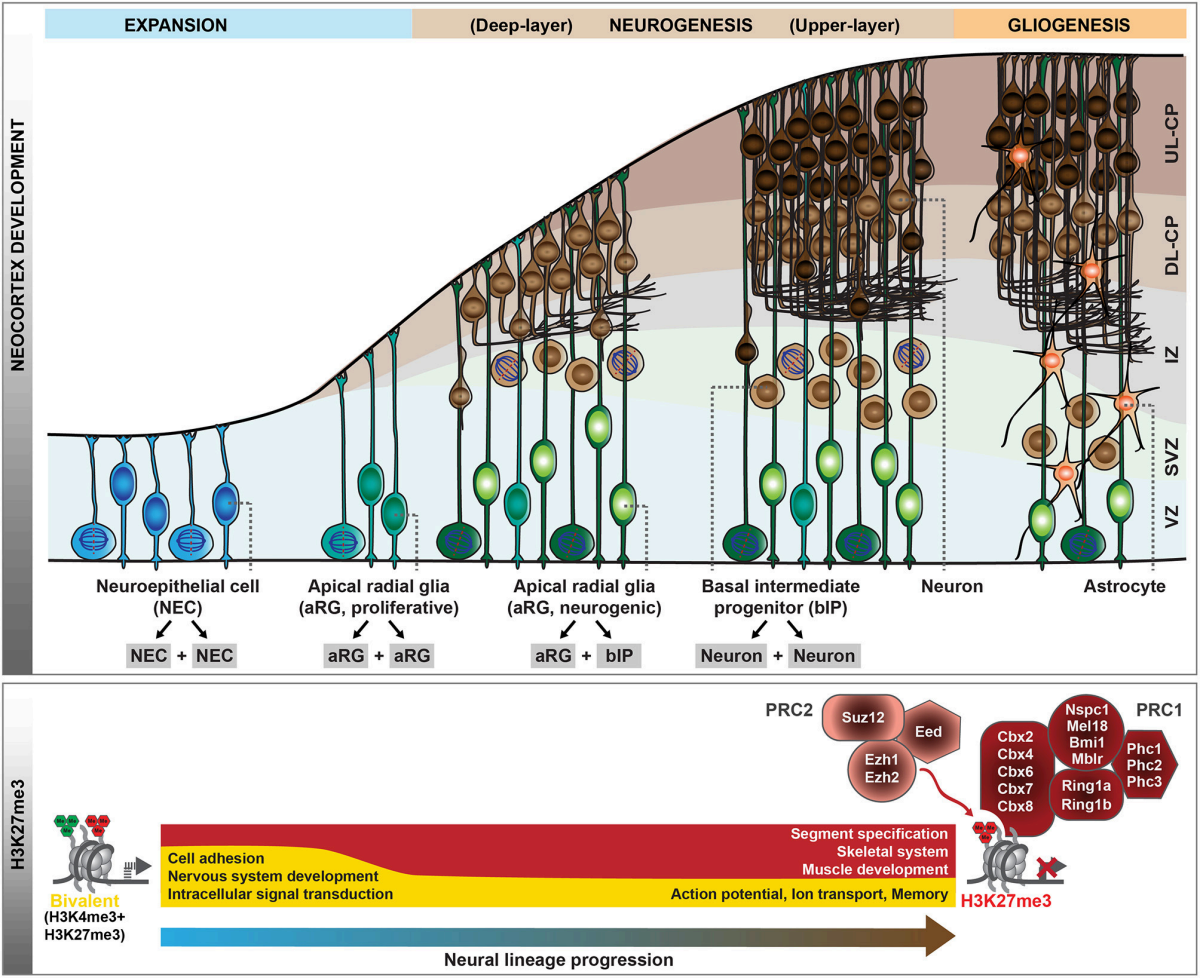
Polycomb-mediated histone methylation during mouse neocortex development. During the development of the mouse neocortex, neural progenitor cells pass through consecutive stages of expansion, neurogenesis, and gliogenesis (top scheme). aRG undergoing neurogenic divisions give rise to bIPs, which are the main source of neurons in the mouse. As neural progenitor cells transition from proliferation to neurogenic divisions, their histone methylation profiles change dynamically. Whereas, many genes are in a bivalent configuration in early proliferative progenitor cells, many of these poised domains are resolved with progressive lineage commitment. The gene ontology categories characteristic of the genes marked by H3K27me3 (red) or bivalent modifications (yellow) during early neurogenesis and in neurons are indicated (bottom scheme). In addition, the core components of Polycomb repressive complex 1 (PRC1) and 2 (PRC2) are shown. VZ, ventricular zone; SVZ, subventricular zone; IZ, intermediate zone; CP, cortical plate; DL, deep-layer; UL, upper-layer.

The generation of neocortical neurons during mouse development is the result of balanced proliferative and differentiative divisions of neural stem and progenitor cells (Götz and Huttner, 2005; Lui et al., 2011; Florio and Huttner, 2014). In the early developing central nervous system, neuroepithelial cells (NECs) function as the primary neural stem cells which show apico-basal polarity and undergo symmetric proliferative divisions to expand the stem cell pool (Figure 1). With the onset of neurogenesis at around mouse embryonic day (E) 10, NECs transform into apical radial glia (aRG), which retain apico-basal polarity and become more elongated. Their cell bodies reside in the ventricular zone, whereas their long basal processes extend to the basal lamina and provide a scaffold for neuronal migration to the cortical plate. aRG are characterized by their ability to self-renew and to simultaneously give rise to neurons, mainly indirectly through basal intermediate progenitors (bIPs). bIPs delaminate from the ventricular surface and reside in the subventricular zone. They lack apico-basal polarity and in mouse typically divide symmetrically to produce two neurons. Neocortical neurons are organized into six horizontal layers, with the deep-layer neurons born first during neurogenesis followed by the generation of upper-layer neurons. At around E17, neurogenesis is terminated and the remaining neural stem and progenitor cells switch to gliogenesis. Thus, throughout mouse neocortical development, the potential of neural progenitor cells (NPCs) for proliferation and differentiation changes as NPCs pass through phases of expansion, deep- and upper-layer neurogenesis, and gliogenesis. In this review, we will discuss the dynamic changes in transcriptional programs and epigenetic information that accompany and guide these transitions. We will mainly focus on post-translational modifications of histones, as the role of other epigenetic pathways, including DNA modifications and chromatin remodeling, in neocortex development are reviewed elsewhere (see Sokpor et al., 2018; Stricker and Gotz, 2018, in this Research Topic).

## Trithorax and polycomb complexes

Epigenetic information, in concert with transcription factors, coordinates the instruction of specific cellular identities from the genomic DNA template, and as such plays an essential role in the transition of cell fates during development. Post-translational histone modifications represent one major epigenetic system, among others. In particular, chromatin modifiers of the Trithorax (TrxG) and Polycomb (PcG) groups were identified as part of an evolutionary conserved epigenetic memory system that acts antagonistically to maintain active and repressed gene expression states, important during stem cell differentiation and embryonic development (reviewed in Piunti and Shilatifard, 2016; Schuettengruber et al., 2017). PcG proteins assemble into two major complexes, PRC1 and PRC2 (Figure 1), which catalyze mono-ubiquitination of histone 2A lysine 119 (H2AK119ub1) and tri-methylation of histone 3 lysine 27 (H3K27me3), respectively. These complexes have also been shown to regulate gene expression during neocortical development, and importantly, are one of the major determinants of the ability of NPCs to either self-renew or to give rise to neurons or glial cells (Tyssowski et al., 2014; Mitrousis et al., 2015; Yao et al., 2016).

## The transition from expansion to neurogenesis

During early development, the neural tube is formed by NECs that divide symmetrically to expand the neural stem cell pool. Following this initial expansion phase, NECs turn into neurogenic aRG, characterized by the appearance of glial hallmarks, a change in the mitotic behavior and a more restricted progenitor fate (Götz and Huttner, 2005; Taverna et al., 2014; Subramanian et al., 2017). This transition is accompanied by a major redistribution of the PcG-mediated H3K27me3 mark (Albert et al., 2017), which is associated with transcriptional gene silencing (Comet et al., 2016). Several tight junction-associated genes convert to a more repressive chromatin configuration, whereas the genes encoding the glial-specific glutamate transporter (*Slc1a3*/Glast) and the brain lipid-binding protein (*Fabp7*/Blbp) acquire H3K4me3 (Albert et al., 2017), a hallmark of TrxG-associated gene activation (Schuettengruber et al., 2017). Notably, in line with NECs representing the earliest and least committed neural stem cells of the developing neocortex, the majority of the genes marked by H3K27me3 in NECs carry H3K4me3 in addition (Albert et al., 2017), a configuration which has been termed “bivalent” (Bernstein et al., 2006). Such bivalent domains are abundant in embryonic and adult stem cells (Shema et al., 2016), where they decorate genes implicated in cell-fate determination and development (Schuettengruber et al., 2017). This has been hypothesized to keep future lineage choices open (Bernstein et al., 2006). With the transition of NECs to aRG, a large fraction of bivalent domains is resolved, either to H3K27me3 at promoters of genes involved in the development of other organs (Figure 1), or to H3K4me3 at genes involved in nervous system development, cell adhesion and cell surface signaling (Albert et al., 2017). Thus, the switch of NPCs from the initial expansion phase to the neurogenic phase is accompanied by major epigenetic changes.

## The neurogenic phase

During the neurogenic phase, aRG have the potential to either proliferate or to self-renew and generate basal progenitors or, rarely, neurons. PcG complexes have been shown to contribute to the regulation of this balance between proliferation and differentiation. The PRC2 histone methyltransferase Ezh2, which generates H3K27me3, is highly expressed in NPCs of the mouse developing neocortex, particularly during early neurogenesis (Pereira et al., 2010; Piper et al., 2014). Specific deletion of *Ezh2* in the developing neocortex from E9.5 results in a loss of H3K27me3 and up-regulation of gene expression, consequently shifting aRG fate from self-renewal toward differentiation (Pereira et al., 2010). This shift results in an overproduction of bIPs and neurons at the expense of aRG, ultimately reducing the neuronal output and leading to a substantially smaller neocortex (Pereira et al., 2010). In light of this, it is interesting to note that the promoters of many transcription factors involved in bIP generation and neuronal differentiation (like *Insm1, Eomes, Neurog1/2*, and *Neurod1/2*) are H3K27me3-positive during the expansion phase of NPCs (Albert et al., 2017), and a loss of this repressive state might contribute to the precocious activation of these genes. In addition, the PRC1 component Bmi1 has been shown to regulate the self-renewal and differentiation of NPCs (Fasano et al., 2007, 2009; Yadirgi et al., 2011).

**Figure 2:**
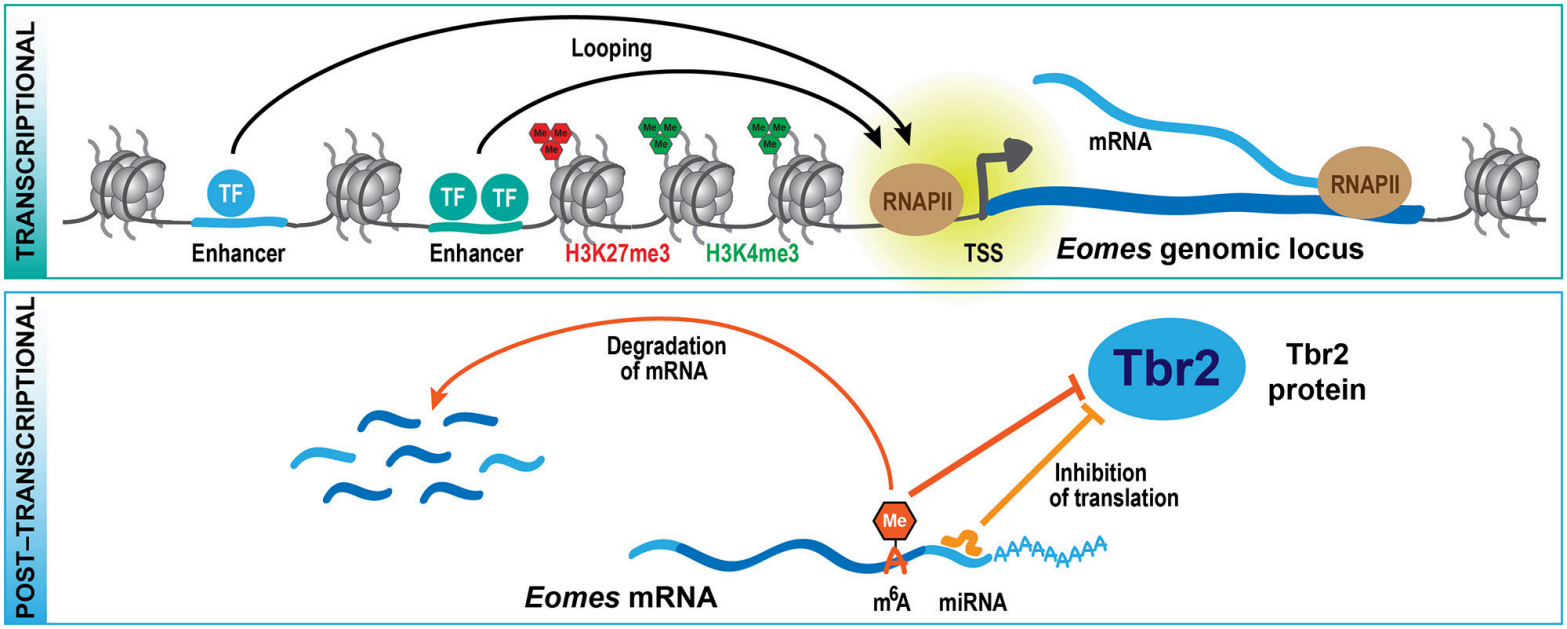
Multi-layered regulation of gene expression. At the transcriptional level (top scheme), cell type-specific expression of genes is regulated by transcription factors that bind to regulatory sequences, including distal enhancers that contact their respective target genes by looping. Histone methylation, mediated by TrxG (H3K4me3) and PcG (H3K27me3) proteins, is part of an epigenetic system that also contributes to the regulation of specific gene expression. At the post-transcriptional level (bottom scheme), the translation and stability of mRNAs is regulated by miRNAs and epitranscriptomic mechanisms including m^6^A, providing a multi-layered system for the control of protein expression during development. These mechanisms are exemplified here for the key transcription factor Tbr2 (*Eomes*), which regulates bIP generation during neocortex development.

From these genetic studies, it is clear that PcG proteins contribute to the regulation of the balance between self-renewal vs. differentiation during neocortex development, but what are the underlying molecular mechanisms? Epigenome profiling in specific cell populations isolated at mid-neurogenesis (E14.5) has shown that H3K4me3 and H3K27me3 marks are highly dynamic during neocortical lineage progression (Albert et al., 2017). In particular, several transcription factors involved in cell fate commitment during neurogenesis display transient changes in histone methylation at their promoters, potentially involved in cell type-specific induction of gene expression. Notably, the promoter of the *Eomes* gene, which encodes the key transcription factor Tbr2 implicated in the generation of bIPs (Arnold et al., 2008; Sessa et al., 2008), changes from a repressive configuration marked by H3K27me3 in proliferative aRG to an active configuration marked by H3K4me3 in aRG undergoing neurogenic divisions (Albert et al., 2017). As these changes likely occur within one cell-cycle, it is conceivable that the H3K27me3 mark is actively removed, most likely by the histone demethylase Jmjd3, which is expressed in the developing neocortex (Sessa et al., 2017) and has been shown to act on *Eomes* gene regulatory regions (Kartikasari et al., 2013). The active configuration of the *Eomes* promoter is largely maintained in bIPs, whereas H4K4me3 levels decline and H3K27me3 is re-established in neurons (Albert et al., 2017), in which *Eomes* is no longer expressed (Florio et al., 2015). Thus, *Eomes* is one example of a gene that undergoes dynamic changes in histone methylation during neocortical differentiation (Figure 2), and these changes correlate well with the gene expression dynamics. In addition, the regulation of other transcription factors that control progenitor proliferation or differentiation has been linked to various histone methylation states, including H3K4me3 and H3K79me3 (Büttner et al., 2010; Yang et al., 2012).

But are the histone methylation patterns instrumental for the correct expression of the related genes in the developing neocortex? Previous studies, which applied CRISPR/Cas9-based genome editing *in vivo* to disrupt *Eomes* expression in NPCs during neocortical development, showed that this acute targeting results in a reduction in bIPs and an increase in neuronal differentiation (Kalebic et al., 2016). Importantly, CRISPR/Cas9-based epigenome editing at the *Eomes* locus in the developing neocortex has shown that the decrease in H3K27me3 in neurogenic NPCs is required for normal Tbr2 expression and bIP regulation (Albert et al., 2017). These results underscore the importance of epigenetic information in the regulation of specific gene expression and as facilitator of cell fate transitions during development.

The H3K27me3 mark is recognized by different “reader” proteins, one of which is the chromatin remodeler Chd5 expressed in neurons of the developing neocortex (Egan et al., 2013). Depletion of Chd5 during neurogenesis results in a block of neuronal differentiation, which can be rescued by Chd5 only if the latter contains an intact double chromodomain mediating H3K27me3 binding. In addition, components of the PRC1 complex can bind to H3K27me3, and at the majority of genomic target sites, H3K27me3 and PRC1 are found to co-localize, even though this traditional model of sequential binding of PRC2 followed by PRC1 complexes has been challenged by several studies (Puschendorf et al., 2008; Blackledge et al., 2015; Kloet et al., 2016). Deletion of Ring1b, an integral component of PRC1 (Leeb and Wutz, 2007), specifically in the mouse developing neocortex during the neurogenic phase results in altered neuronal subtype specification (Morimoto-Suzki et al., 2014). By mediating the timed termination of *Fezf2* expression, Ring1b regulates the number of subcerebral projection neurons produced. These data suggest that PcG complexes and associated proteins control several aspects of cortical neurogenesis, including the balance between self-renewal and differentiation of aRG as well as the switch from deep- to upper-layer neurogenesis in NPCs.

## The transition to the gliogenic phase

In mouse, the neurogenic phase is followed by a period of gliogenesis, during which astrocytes and oligodendrocytes are generated. The timing of the switch from neurogenic to gliogenic fate of NPCs is critical for brain development, as it is one of the determinants of the final number of cortical neurons produced. In addition to extracellular cues, cell-intrinsic programs regulate NPC fate, to which epigenetic mechanisms are thought to contribute. The PcG proteins have been demonstrated to play an important role in the timing of the neurogenic to gliogenic transition. Depletion of PcG proteins during the neurogenic period leads to a prolonged neurogenic phase of NPCs and a delayed onset of astrogenesis (Hirabayashi et al., 2009; Sparmann et al., 2013). Toward the time when neurogenesis is normally terminated, several genes associated with the neurogenic lineage are selectively derepressed in PcG-mutant NPCs, including neurogenin 1 (*Neurog1*), a key proneural transcription factor that can suppress astrocytic differentiation (Hirabayashi et al., 2009).

Interestingly, deletion of Ezh2 before, or at, the onset of neurogenesis has the opposite effect, leading to a shorter neurogenic period and precocious astrocyte generation (Pereira et al., 2010; Sparmann et al., 2013). In NPCs *in vitro*, PcG proteins mediate the suppression of the key astrogenic marker *Gfap* (Mohn et al., 2008; Sparmann et al., 2013), which has been proposed to prevent the premature onset of gliogenesis (Sparmann et al., 2013). In the developing neocortex, however, the promoters of *Gfap* as well as of other genes characteristic of astrocytes are not marked by H3K27me3 at mid-neurogenesis (ENCODE Project Consortium, 2012; Albert et al., 2017), which is in agreement with other reports suggesting a role for alternative repressive pathways, including DNA and H3K9 methylation, in the regulation of astrocyte-specific genes (Takizawa et al., 2001; Song and Ghosh, 2004; Fan et al., 2005; Hatada et al., 2008). Future research should be aimed at identifying PcG target genes underlying the context- and stage-dependent role of PcG proteins in NPCs during different phases of neocortex development, and should provide a more general view beyond the limited number of well-characterized known regulators.

## Cell type- and stage-specific roles of polycomb proteins

Previous studies in mouse and human embryonic stem cells (Mikkelsen et al., 2007; Mohn et al., 2008; Burney et al., 2013; Ziller et al., 2015) and the developing mouse neocortex (Albert et al., 2017) have shown that H3K27me3 levels are highly dynamic at different NPC stages and during neuronal differentiation, raising the question of how PcG target gene specificity is achieved. One way to dynamically control PcG function is by altering the composition of PcG complexes, which in mammals, as opposed to flies, is highly diverse, enabling the assembly of various sub-complexes with different chromatin binding affinities and interaction partners (Piunti and Shilatifard, 2016; Schuettengruber et al., 2017).

In embryonic stem cells, the interchange of Chromobox (Cbx) family proteins, part of PRC1, has been reported to modulate the balance between self-renewal and lineage commitment (Morey et al., 2012; O'Loghlen et al., 2012; Santanach et al., 2017), and different Cbx paralogs are required for different cell lineages (Luis et al., 2011; Klauke et al., 2013). Of note, the Cbx paralogs are differentially expressed in neural sub-populations of the developing neocortex (Florio et al., 2015). Moreover, chromatin remodelers of the chromodomain helicase DNA-binding (Chd) family, which have been reported to interact with PcG complexes, also show differential expression during neocortex development. Whereas Chd5 is expressed in neurons and controls neuronal differentiation (Egan et al., 2013), Chd4 is expressed in NPCs during early neurogenesis where it has been proposed to function in PcG-mediated inhibition of astroglial differentiation (Sparmann et al., 2013). This switch in subunit composition may contribute to the re-targeting of PcG complexes during neocortex development.

PcG complexes themselves bind relatively unspecifically to CG-rich regions lacking DNA methylation (Schuettengruber et al., 2017). In addition, the chromatin targeting of PRC2 is stabilized by interactions with transcription factors, non-coding RNAs and other chromatin factors resulting in increased binding and H3K27me3 deposition at specific regions. The highly restricted expression pattern of many of these factors and RNAs during neocortex development (Aprea et al., 2013; Molyneaux et al., 2015; Liu et al., 2016) provides a potential mechanistic explanation for cell type-specific PcG targeting. Moreover, the H3K27me3-specific histone demethylase Jmjd3 has been implicated in the activation of neuronal gene expression (Jepsen et al., 2007; Park et al., 2014), and associates with the transcription factor Tbr2 in the developing neocortex (Sessa et al., 2017), further contributing to the dynamic regulation of H3K27me3.

## Transcriptional pre-patterning

During recent years, there have been massive efforts to characterize the transcriptomic signatures of the various NPC subtypes in the mouse developing neocortex, but also in other mammalian species including the ferret, macaque and human (Ayoub et al., 2011; Fietz et al., 2012; Aprea et al., 2013; Arcila et al., 2014; Miller et al., 2014; Pollen et al., 2014; Camp et al., 2015; De Juan Romero et al., 2015; Florio et al., 2015; Johnson et al., 2015; Liu et al., 2016; Telley et al., 2016; Nowakowski et al., 2017; Zhong et al., 2018). From these studies, a variety of gene expression differences have been uncovered that underlie specific cell biological features, proliferative capacities and differentiation potential of the distinct NPC types (reviewed in Silver, 2016; Florio et al., 2017). Interestingly, several of these studies described the expression of genes in aRG whose protein products are well-known to function only downstream in the lineage, in bIPs or neurons (Florio et al., 2015; Telley et al., 2016; Nowakowski et al., 2017), raising the possibility that there is a delay in translation for certain mRNAs.

One example of such a gene that is expressed already in aRGs, specifically those undergoing neurogenic divisions, is *Eomes* (Florio et al., 2015), which gives rise to the bIP transcription factor Tbr2 (Arnold et al., 2008; Sessa et al., 2008). What is it that keeps the *Eomes* mRNA from being translated in aRG? The Tbr2 protein has been shown to be repressed by the microRNAs (miRNAs) miR-92 and miR-92b, and both miRNAs regulate bIP specification in the developing neocortex (Bian et al., 2013; Nowakowski et al., 2013). Interestingly, miR-92 and miR-92b are specifically expressed in aRG undergoing neurogenic divisions, where the *Eomes* mRNA is highly expressed (Florio et al., 2015). In contrast, bIPs, which express Tbr2 protein, have low levels of both miRNAs (Nowakowski et al., 2013; Florio et al., 2015). Of note, many other miRNAs display unique profiles of expression in the developing neocortex (Barca-Mayo and De Pietri Tonelli, 2014; Rajman and Schratt, 2017), and among their validated target genes are several cell cycle and neurogenesis regulators (Arcila et al., 2014; Fei et al., 2014), indicating that miRNA-mediated control of RNA translation (Figure 2) may play a widespread role during neocortex development and also evolution. Moreover, two components of the miRNA microprocessor complex, Drosha and DGCR8, were shown to regulate gene expression in the developing neocortex in a miRNA-independent fashion (Knuckles et al., 2012; Marinaro et al., 2017), further adding to the complexity of post-transcriptional gene regulation.

In addition, recently a new epitranscriptomic mechanism has been identified that regulates the metabolism and translation of mRNAs, which involves the post-transcriptional modification of mRNAs by *N*^6^-methyladenosine (m^6^A) (reviewed in Zhao et al., 2017). Depletion of m^6^A during neocortex development leads to a prolonged cell cycle of aRGs and extends neuron production to postnatal stages, suggesting that m^6^A regulates cortical neurogenesis (Yoon et al., 2017). Among the transcripts that are tagged by m^6^A, several encode transcription factors regulating NPC fate, such as Pax6, Sox2, Neurog2, and Tbr2. The presence of m^6^A on these transcripts promotes their rapid turnover, and in absence of the m^6^A methyltransferase complex component *Mettl14*, several neuronal lineage proteins, like Neurod1 and Tbr2, are precociously expressed in aRG. This observation led to the proposal of the novel concept of transcriptional pre-patterning during cortical neurogenesis, whereby a subset of neuronal lineage genes is already expressed in aRG but their levels actively suppressed post-transcriptionally by m^6^A-dependent decay (Yoon et al., 2017). A second study that analyzed the role of m^6^A during neurogenesis found that *Mettl14* deletion results in decreased radial glia proliferation and premature differentiation (Wang et al., 2018). The authors of this study ascribed the observed phenotypes to genome-wide changes in histone methylation patterns which may result from the destabilization of transcripts that encode histone-modifying enzymes. While further mechanistic studies are required to dissect the role of m^6^A in specific neural subpopulations, the two studies (Yoon et al., 2017; Wang et al., 2018) describe a novel post-transcriptional mechanism regulating protein expression during neurogenesis (Figure 2).

## Epigenetic pre-patterning

Whereas, transcriptome analysis provides a snapshot of a cell's gene expression pattern at a specific point in time, the corresponding epigenetic information captures gene regulatory mechanisms, developmental origins, and potential future responses to developmental stimuli (Mo et al., 2015). Transcription factors, which are thought to be instrumental for the specification of cell type-specific gene expression programs, bind to DNA in the context of chromatin, which carries multiple post-translational modifications, and these affect transcription factor binding (Shlyueva et al., 2014; Yin et al., 2017). As such, the epigenetic landscape can permit the transcription of certain genes, while rendering others inaccessible to most transcription factors.

That said, the transition from “closed” to “open” chromatin, and vice versa, is determined by regulatory proteins, most prominently a special class of transcription factors, called pioneer factors (Shlyueva et al., 2014). These factors can bind to repressed chromatin and recruit chromatin remodelers to evict nucleosomes to open up the region, thereby making the DNA accessible to other transcription factors. During neural differentiation, such pioneer factors have been proposed to remodel the binding site repertoire for proneural factors at the NPC stage by changing the epigenetic landscape at their respective target sites (Ziller et al., 2015). This is also thought to ensure proper further lineage specification by restricting the binding capacity of proneural and other transcription factors toward appropriate sites.

Differential gene expression in specific cell types is mainly controlled by distal *cis*-regulatory elements, among which enhancers are the most abundant (Spitz and Furlong, 2012; de Laat and Duboule, 2013). Enhancer sequences contain short DNA motifs that serve as binding sites for sequence-specific transcription factors. In a given tissue, active enhancers are brought into spatial proximity of their respective target gene by looping (Shlyueva et al., 2014). Our understanding of how chromatin is organized and folded within the nucleus, and how this affects gene regulation and cell fate decisions, has greatly expanded during recent years, mainly due to technological advances in detecting chromatin contacts in 3D (Bonev and Cavalli, 2016; Franke et al., 2016).

During neural differentiation, both *in vitro* and in the mouse developing neocortex, chromatin interactions change dynamically, frequently related to neural transcription factors that contribute to chromosome reorganization (Bonev et al., 2017). In addition, PcG proteins have been proposed to facilitate neural induction by establishing physical interactions between poised enhancers and their target genes in embryonic stem cells (Cruz-Molina et al., 2017). These preformed contacts are thought to provide a permissive topology that facilitates the timely and robust induction of major neural genes upon differentiation. The importance of understanding chromosome conformation has been underscored by recent studies in the human developing neocortex, which have revealed regulatory relationships relevant to the evolution of human cognition but also to diseases (Won et al., 2016; de la Torre-Ubieta et al., 2018).

## Conclusions

It is well-established that epigenetic mechanisms contribute to the regulation of gene expression during stem cell differentiation and development. In this review, we have summarized recent advances in our understanding of the role of Polycomb proteins during mouse neocortex development. In particular, recent epigenome profiling has shed further light on the context-dependent functions of Polycomb proteins during the proliferative and neurogenic phase of neocortex development. It remains to be shown on a genome-wide scale how PcG targets change with the transition to the gliogenic phase. Moreover, in the future, it will be interesting to apply the emerging CRISPR/Cas9-based epigenome editing tools (Pulecio et al., 2017) to dissect the role of epigenetic changes at gene regulatory regions of important regulators of neocortex development. In a proof of principle study, the role of H3K27me3 has been analyzed *in vivo* during neocortex development at the *Eomes* gene promoter (Albert et al., 2017). From such epigenome editing experiments, further functional insights into chromatin-mediated gene regulation can be expected. Importantly, such studies will allow to move the field forward beyond correlations of epigenetic information and gene expression to interrogating the functional relevance of histone modifications at regulatory regions in specific neural cell types and at various periods of neocortex development. Recent technological advances have revealed important insights into the 3D genome organization during neocortex development and have led to the identification of distal regulatory elements. With CRISPR/Cas9-based genome and epigenome editing techniques at hand, the functional interplay of histone modifications, genome organization, and gene expression can now be unraveled.

The epigenetic landscape provides a framework within which many transcription factors operate, but which, in turn, is modulated by the action of transcription factors and gene expression itself. During development, epigenetic patterning is important for the correct spatio-temporal regulation of gene expression. In addition, the translation of expressed mRNAs is regulated by miRNAs and novel epitranscriptomic modifications, providing a multi-layered mechanism to precisely control the dynamic expression of genes, both at the mRNA and protein level. The challenge for the future will be to integrate the different layers of transcriptional and post-transcriptional gene regulation into a comprehensive framework that allows to link the different mechanisms and to understand the cross-talk between these systems.

## Author contributions

MA designed the figures. MA and WH wrote the manuscript.

### Conflict of interest statement

The authors declare that the research was conducted in the absence of any commercial or financial relationships that could be construed as a potential conflict of interest.
